# A Forensic Case of Suicide Ingestion of Paraquat Herbicide: New Histological Insights and Revision of the Literature

**DOI:** 10.1097/PAF.0000000000000878

**Published:** 2023-09-20

**Authors:** Stefano Tambuzzi, Laura Vacchiano, Guendalina Gentile, Michele Boracchi, Riccardo Zoja, Arnaldo Stanislao Migliorini

**Affiliations:** From the ∗ Laboratory of Forensic Histopathology and Forensic Microbiology, Institute of Forensic Medicine, Department of Biomedical Sciences for Health, University of Milan; † School of Medicine, Vita-Salute San Raffaele University, Milan, Italy.

**Keywords:** forensic pathology, paraquat, suicidal ingestion, caustic substance, oxidative stress, forensic autopsy

## Abstract

Paraquat (PQ) is one of the most widely used herbicides in the world, and poisoning is generally associated with accidental, suicidal, or homicidal events. Therefore, in the forensic context, PQ could be in various ways involved as a possible cause of death of a subject. However, even though its systemic toxicity is known, the biological effects exerted on individual viscera have been explored only to some extent, especially in case of victim's survival. Therefore, a case concerning a suicidal ingestion of PQ with survival of 3 days was deemed of interest. Clinical toxicological analyses confirmed acute PQ intoxication, and after the death of the victim, an autopsy was performed showing local and systemic signs of ingestion of a caustic substance. Histologic examination revealed marked cellular damage to the major viscera, particularly the gastroesophageal tract, liver, kidneys, and lungs, with initial alveolar fibrosis noted despite the patient's short survival. This finding represents a new element in the context of PQ lung injury, as it has not been previously documented in the literature. Thus, histological findings in lethal intoxications after survival can reveal specific peculiarities still unknown and, therefore, assume transversal relevance not only at forensic but also clinical level.

International literature attributes 14% to 20% of total suicides to agricultural pesticide poisoning,^1^ making them one of the main contributors to the global suicide burden.^2^ Among all agricultural pesticides, Paraquat (PQ) is one of the most widely used herbicides in the world, and poisoning is generally associated with accidental events,^3,4^ suicides,^5–7^ or homicides.^8–13^ Accidental occurrences^14,15^ are usually detectable a few hours after exposure and occur mainly among agricultural operators. They are due to noncompliance with the precautions of use of PQ or negligence in its custody or handling. In particular, its careless transfer into inadequate containers, not properly labeled and guarded, plays a main role, with consequent exchange of the toxic substance for beverages. Skin absorption is poor if exposure is short, the skin is intact, and the surface contact is limited to small areas. Significant absorptions occur through contact with large abraded or deepithelized skin surfaces and in case of prolonged adhesion of PQ-impregnated clothing, perhaps as a result of accidental leakage from containers carried on the shoulders of agricultural operators.^14,15^

Paraquat suicides have been reported as “alarming” in some rural contexts, overcoming homicidal and accidental exposures, where they represent the second cause of poisoning^2,16^: the subjects involved voluntarily ingest large quantities of the herbicide that rapidly exerts a high intrinsic local irritant toxicity, with systemic symptoms that appear later. Literature also describes some rare cases of parenteral injections of PQ for suicide purposes.^5,17,18^

Homicidal PQ poisonings are rare, generally occult,^13^ and initially very difficult to diagnose, especially if exposure is not clearly denotable or symptoms are mild and nonspecific. This may lead to a diagnostic delay due to nonrecognition of poisoning and the implementation of nonspecific procedures as recommended by both guidelines and good clinical practices. Because these actions are planned, administration of multiple low doses of pesticide are often observed^19^ in which the burning taste of PQ can be masked by mixing it with hot liquids, spicy foods, or alcohol.^8^ In some cases, preliminary stunning of the victims by asphyxiation is reported in literature with the purpose of forcing the ingestion of PQ by pouring the substance directly into the throat.^12^ It is therefore evident that, in a forensic context, it is far from unusual to come across scenarios in which PQ is in various ways involved as a possible cause of death of a subject. Depending on the type of exposure and the amount of herbicide ingested, the spectrum of resulting biological signs can vary greatly in severity and lethality. Such manifestations have been described in literature, but if systemic toxicity of PQ is known, the induced histopathological lesions have been explored only to some extent, especially in case of victim's survival. Therefore, starting from a case of our direct observation of acute lethal PQ intoxication, we present the microscopic evidence that emerged, associating a complete and broad iconography and discussing the forensic aspects.

## THE CASE

A 31-year-old man recently diagnosed with major depressive disorder, while in a hotel room, called for help and referred to the rescuers, once arrived, that he had purchased a 1-L container of pesticide from a farm chemical supplier and had ingested some sips for suicide purposes about 5 hours before. After the appearance of worsening illnesses, however, he reported that he was frightened and therefore wanted to seek help. To confirm this, an empty plastic bottle bearing the words “Gramoxone W” was found on the floor of the room, a 20% liquid formulation of PQ with a concentration of 200 g/L. The man was hospitalized with symptoms of marked asthenia and nausea, sour vesicular murmur, cough, difficulty swallowing and pharyngodynia, blood pressure of 130/70 mmHg, heart rate of 130 beats per minute, and respiratory rate of 26 acts/min. There was no history of any comorbidities, and the man was not following any pharmacological therapy. Clinical toxicological investigations, performed on the patient, measured 1235 ng/mL of PQ in blood and 287,000 ng/mL in urine. Acute renal failure (urea, 27.3 mmol/L; creatinine, 452 mmol/L) was also diagnosed and treated via dialysis while PQ intoxication therapy was initiated with steroids, cyclophosphamide, and acetylcysteine.

Esophagus-gastro-duodenoscopy showed marked edema and hyperemia of the mucous membrane of the hypopharynx, as well as multiple deepithelized areas to the esophageal mucosa with a picture of III degree esophagitis. Despite all the care provided, a marked impairment of the respiratory function could be noted, characterized by hypoxia and hypercapnia, and addressed with the patient's intubation and the use of positive end-expiratory pressure mechanical ventilation. The onset of right pneumothorax required thoracic drainage. In the 2 days after hospitalization, blood levels of PQ (Table 1) remained elevated. The substance was measured several times via high-performance liquid chromatography–tandem mass spectrometry technique. Toxicological analyses for other substances of toxicological interest resulted to be negative.

**TABLE 1:** Values of Paraquat Quantitated Intravitam in the Victim's Body Fluids


Substrate	Day of Withdrawal	Sample Collection Time	PQ concentration, ng/mL	
Serum	I day of hospitalization	16.30	1235	
Urine	I day of hospitalization	16.30	287,000	
Serum	II day of hospitalization	06.00	1290	
Serum	II day of hospitalization	14.00	1210	
Serum	III day of hospitalization	08.00	1870	

The patient was then seized by psychic agitation, tachycardia, hypotension, fever, and progressive hypoxia. A multiple-organ-failure syndrome (acute respiratory distress syndrome, renal failure, hepatic failure, rhabdomyolysis) with severe hypoxia and hypercapnia and marked impairment of lung compliance occurred. The administration of amines (dopamine, adrenaline) was initiated to support the cardiovascular system. However, the onset of ventricular tachycardia, followed by bradycardia and cardiac arrest unresponsive to resuscitation procedures, led to the death of the patient. Overall, the exitus occurred 3 days after the ingestion of the toxic substance. A judicial autopsy was ordered by the prosecutor to identify the cause of death 48 hours after the deceasing.

### The Autopsy

**FIGURE 1:**
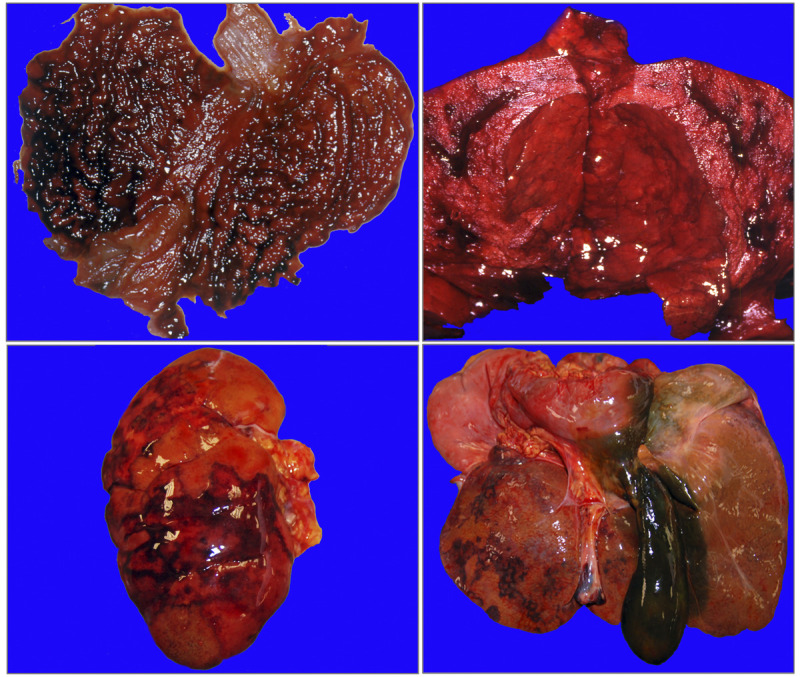
Autopsy visceral macroscopic pictures: in A, stomach with evident areas of hemorrhagic infiltration of the mucosa; in B, lungs with paler parenchyma areas alternating with other partially confluent red-purple areas; in C, left kidney with cortical layer characterized by multiple and small purpuric areas; in D, liver with focal hemorrhagic superficial areas and gallbladder containing dense blackish bile.

Upon autopsy, the body was in an excellent state of preservation; it weighed 82 kg and was 178-cm long. The external examination of the corpse, in addition to the signs of hospitalization, showed only extensive and irregular areas of peribuccal skin loss and lips with parchment drying, brownish in color. Internal examination showed hemorrhagic punctuations on the dorsal surface of the tongue and epiglottis, as well as an esophagus with thin walls, diffusely reddish and deepithelialized mucosa. In the stomach (Fig. 1A) and duodenum, there were areas of hemorrhagic infiltration of the mucosa with focal areas of detachment of the membrane. The pleural cavities contained a total of about 1 L of serum-blood fluid. The lungs (Fig. 1B) appeared heavier (1230 g the left, 1350 g the right), purplish, hard-elastic, and with multiple subpleural hemorrhagic suffusions spread to all lobes, bilaterally; at the cut, paler parenchyma areas emerged alternating with other partially confluent red-violet areas, more frequent in the peribronchial region. The kidneys (Fig. 1C) (right, 87 g; left, 68 g) were congested and smaller than normal; moreover, the cortical layer was thinner than usual with multiple and small purpuric areas, whereas the medulla showed larger and paler areas. Other gross findings included (1) cerebral edema, associated with flattened sulci and circumvolutions if compared with a regularly conformed organ (brain: 1033 g); (2) punctiform subepicardial hemorrhagic suffusions arranged mainly on the diaphragmatic surface (heart, 347 g); (3) yellowish-brown congested liver (Fig. 1D) (1213 g), with focal hemorrhagic superficial areas deepening into the parenchyma and gallbladder containing dense blackish bile; (4) pancreas with reddish areas of hemorrhagic appearance, with irregular and blurred margins; and (5) bladder with thickened walls and fibrotic consistency, with a mucosa characterized by hemorrhagic punctuation. All the viscera, with the exception of the lungs, appeared softer in consistency. At the end of the gross examination, only histological investigations were authorized because multiple toxicological analyses had already been performed intravitam.

### Histological Analyses

**FIGURE 2:**
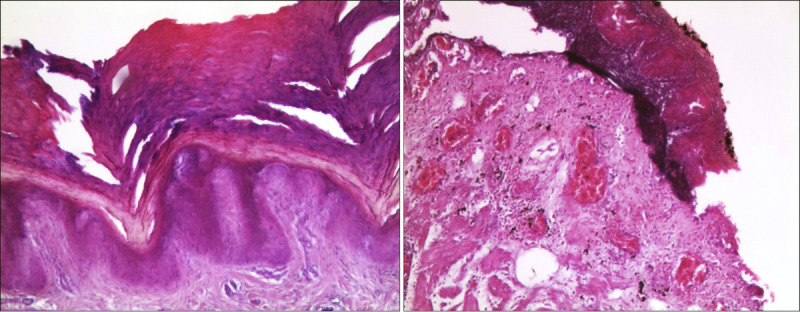
Microscopic pictures in H&E: in A, peribuccal skin with areas of detachment and ulceration (100×); in B, esophageal mucosa with necrotic-hemorrhagic lesions, ulcerations, and inflammatory infiltrates (100×). H&E, hematoxylin and eosin.

**FIGURE 3:**
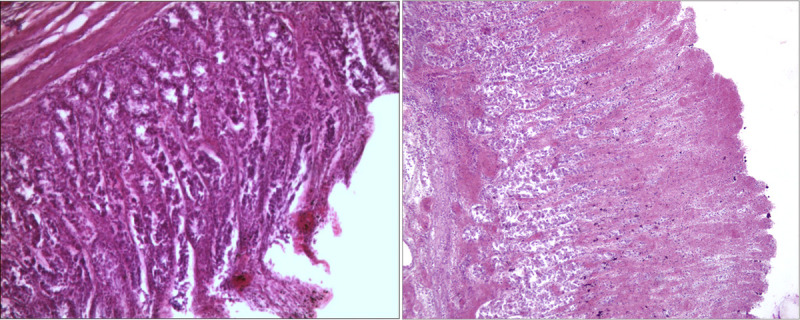
Microscopic pictures in H&E: gastric (A) and intestinal mucosa (B) with necrotic-hemorrhagic lesions and acute hemorrhagic infiltrates with areas of mucosal detachment (100×). H&E, hematoxylin and eosin.

Standard postfixative histopathologic examination was performed on a sample of peribuccal skin and on viscera fragments (brain, esophagus, heart, lungs, stomach, liver, pancreas, spleen, intestine, kidneys, and bladder) that had been collected during the autopsy examination. Histological sections of the brain showed edema and vascular congestion. Necrotic-hemorrhagic lesions emerged in the peribuccal skin samples (Fig. 2A) and esophageal mucosa (Fig. 2B), with several superficial ulcerations partly under repair and inflammatory infiltrates in the esophageal submucosa. Necrotic-hemorrhagic lesions were also observed in the gastric wall (Fig. 3A) and duodenum (Fig. 3B), with detachment of focal areas of mucosa and inflammatory submucosal infiltrates.

**FIGURE 4:**
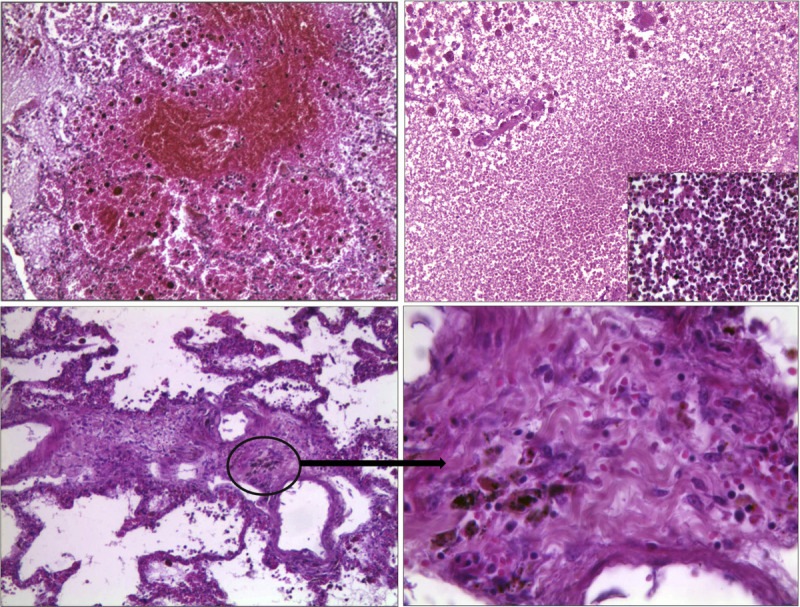
Microscopic pulmonary pictures with H&E: in A, massive areas of pulmonary hemorrhage with alveolar flood, in the presence of severe edema (100×); in B, almost total destruction of the alveolar septa (200×), in presence of massive inflammatory infiltrate, as shown in the detail (400×); in C, alveolar septa thickened (100×), with detail of fibrosis and fibroblasts at higher magnification in D (400×). H&E, hematoxylin and eosin.

The lungs appeared edematous (severe degree) with the presence of inflammatory and hemorrhagic infiltrates in the alveoli and septa and with the presence of hyaline membranes; there were also areas of alveolar destruction and foci of abscessualized pneumonia. In addition, initial fibrosis was observed at the alveolar septa, which appeared thickened in the presence of inflammatory infiltrates arranged in small irregular aggregates (Fig. 4).

**FIGURE 5:**
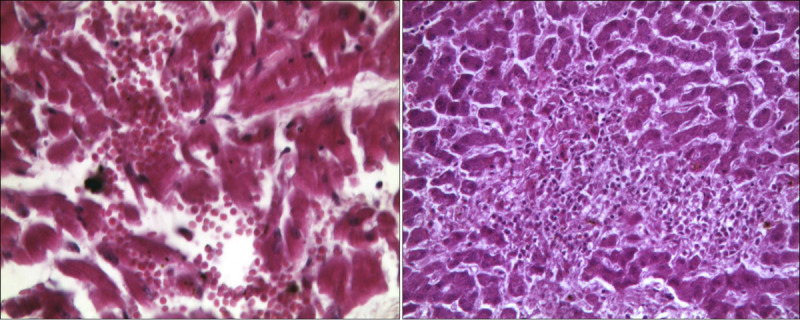
Microscopic pictures in H&E: in A, heart with evidence of multiple foci of interstitial hemorrhage (400×); in B, liver with diffuse foci of hepatocellular necrosis (400×). H&E, hematoxylin and eosin.

**FIGURE 6:**
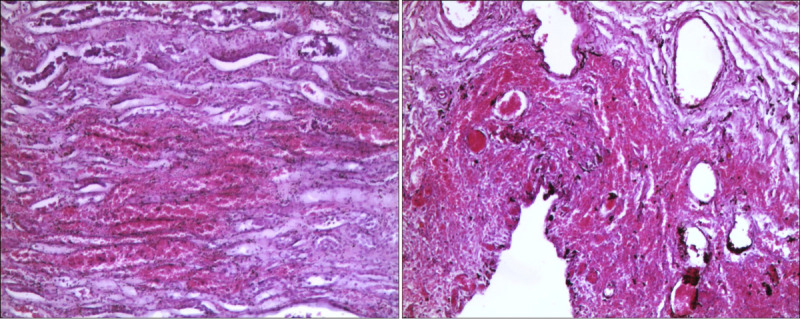
Microscopic pictures in H&E: in A, kidneys with evidence of multiple and diffuse hemorrhagic infiltrates and acute tubular necrosis in the context of inflammatory infiltrate (100×); in B, detail of bladder mucosa with marked hemorrhage (100×). H&E, hematoxylin and eosin.

Multiple interstitial hemorrhagic foci and nonspecific signs of cardiac distress (waves, thinned fibers, shrinkage band necrosis) could be documented in the myocardium (Fig. 5A). In liver samples, foci of hepatocellular necrosis and mild inflammatory infiltrates were observed (Fig. 5B). Kidneys showed signs of acute renal damage, with glomerular, tubular, and interstitial hemorrhagic infiltrates, as well as acute inflammation and foci of acute tubular necrosis (Fig. 6A). Finally, a picture of hemorrhagic cystitis emerged (Fig. 6B).

At the end of the performed investigations, autopsy and histological findings, in association with intravitam toxicological findings, allowed to attribute the death to a consequence of multiple-organ-failure secondary to acute intoxication from oral ingestion of PQ.

## DISCUSSIONS

Paraquat (C_12_H_14_N_2_C_l2_; *N*,*N*′-dimethyl-4,4′-bipyridinium dichloride; CAS:1910-42-5) is a potent herbicide/desiccant belonging to the dipyridyl herbicide compounds. Paraquat in the form of pure salt is crystalline, white, and odorless; technical products are yellow, whereas aqueous solutions are red. Identified as methyl viologen in 1932 and used as a colorimetric indicator of oxidation-reduction, it has had a profound economic, social, and agronomic impact on agricultural crops and pest control gardening^20^ in over 120 developed and developing countries^21^ after the discovery of its herbicidal properties. The intrinsic toxicity of PQ, its caustic action on biological tissues, and, at the same time, the lack of effective treatments^22^ make related poisoning one of those with the highest mortality in the toxicological field,^23^ even if it is ingested in minimal doses.^24^ Paraquat can be absorbed by skin contact leading to erythema, peeling skin, flittene, necrosis, hemorrhages, and nail flaking up to nail fall.^25^ Another manner of absorption is via inhalation of droplets or nebulized dust, causing damage to the nasal and pharyngeal mucous membranes, epistaxis, pharyngodynia, asthma, vomiting, and headache; frequently, ocular damage is associated with exfoliation of the corneal and conjunctival epithelia, corneal edema, eye pain, photophobia, and decreased visual acuity. Oral ingestion is the most fatal modality,^26^ and the estimated minimum lethal dose for men is approximately 35 mg/kg of body weight, although a smaller amount can still be lethal without timely treatment.^12^ In particular, the ingestion of liquid formulations containing 20% of PQ, such as Gramoxone and Dextrone, are associated with a mortality of approximately 60% with a minimum lethal dose that has been estimated to be 10 mL.^27^

The effects of ingestion are dose dependent^26^: (1) in the ingestion of up to 2 g of PQ, a typical biphasic evolution shows, in which, after 2 or 3 days from the intake, the oropharyngeal and gastrointestinal manifestations tend to regress, whereas the first signs of pulmonary involvement tend to appear, usually followed by death within 2 to 6 weeks; (2) in the ingestion of 3 to 6 g of PQ, mucosal lesions appear within 24 hours followed by irritation and worsening caustication of the gastrointestinal tract with possible perforations of the esophagus leading to mediastinitis and stomach, liver, and kidney damage that can be followed by pulmonary manifestations leading to deceasing of the patient usually within 5 to 7 days; (3) gastroesophageal and cardiac signs, metabolic acidosis, and death within 24 hours due to multiorgan failure, cardiovascular abnormalities and collapse, or severe respiratory problems occur immediately upon ingestion of more than 6 g of PQ.^25^

It is believed that PQ is not significantly biotransformed in the human body, concentrating within many cells. The crucial phase of PQ's mechanism of action is a cyclic and repetitive oxide/reduction of a single electron of the molecule of origin that causes, in a biological system, 2 relevant consequences for the development of the molecule's toxicity. The first concerns the production of activated oxygen species (eg, superoxide anion, hydrogen peroxide, hydroxyl radical) that are extremely reactive with tissue components in which they cause direct damage and formation of other reactive oxygen species and nitrite radicals. The second concerns the depletion of tissue reducing equivalents (eg, NADPH) among the main antioxidant defenses of cells, necessary for their normal functioning.^28^ Oxidative stress created by free radical production and NADPH depletion can directly cause cellular injury through mitochondrial dysfunction and lipid peroxidation with oxidative degeneration of membranes' polyunsaturated fatty acids,^28–30^ triggering a pronounced secondary inflammatory response. Also cell death may occur through either necrosis or apoptosis.

In a variable period of hours/days and with biphasic distribution, these processes affect organs with high blood flow, oxygen tension and energy requirements, in particular lungs,^29^ heart,^31^ kidneys,^32^ and liver^26^ with multiple organ deterioration. Regarding the nervous system, PQ does not readily cross the blood-brain barrier, so the brain is less commonly affected, although this substance has also been detected in cerebrospinal fluid. The generation of reactive oxygen species and mitochondrial damage are neurotoxic factors, and recent studies have established a relationship between PQ and Parkinson disease.^33^ The primary and extensive damage caused by PQ is typically concentrated in the lung^34^ where the xenobiotic is actively absorbed against a concentration gradient, accumulating in Clara epithelial cells and in types I and II alveolar cells.^22^ In these cells, the early destructive phase is followed by a proliferative one, in which the molecule causes alveolitis, edema, and infiltration of inflammatory cells with pneumonia and pulmonary fibrosis.^35^

The case presented concerned an acute PQ poisoning by oral ingestion of this toxic substance for suicide purposes, as testified by the victim's own admission. Upon entering the hospital, severe intoxication was demonstrated, as clinical toxicological tests revealed very high blood levels of Paraquat. They remained almost constant and, in any case, always above 1200 ng/mL for the 3 days of survival, despite hospitalization and treatment. In fact, the intoxication rapidly evolved into a severe and irreversible syndrome of multiple-organ failure that was, at last, fatal. Consistently, at autopsy examination, clear findings of a gastrointestinal transit of an irritative substance, such as PQ, emerged, with deepithelialization and necrosis of the mucous layer; on the other hand, systemic signs included severe pulmonary and hepatorenal impairment. Histologically, the involvement of the main organs, in particular of the lungs, was confirmed and evaluated as consistent with the data reported in literature.^36^ Severe edema, hyaline membranes, pulmonary inflammation, hemorrhages, and pneumonia foci could be noted, which fully justified the severe respiratory failure, which rapidly manifested itself and continued throughout hospitalization. However, considering the short survival time of the victim (3 days only) after ingestion of PQ, it was particularly interesting to note initial pulmonary fibrosis of the alveolar septa. In literature, fibroblast deposition and collagen production are usually described as occurring 5 to 8 days after the injury. However, it is known that, in particularly severe damages, early fibroblast appearance can occur in about 1 day.^37^ In the specific case of PQ, there is immediate destructive alveolitis because of accumulation in the alveolar cells of the lungs.^38^ This is a very important pattern of damage that can be followed by a rapid proliferative phase with fibroblast deposition and the appearance of early fibrosis. This could be considered as evidence of the strong damaging effect that PQ exerts on the lungs when large amounts are ingested. Overall, therefore, despite the short survival time, an initial but not negligible early picture of alveolar fibrosis was observed, which did not develop further because of the death of the victim.

Further histological findings consisted of esophagogastric, hepatocyte, and tubular acute necrosis, demonstrating the picture of multiorgan failure that accompanied lung damage. There were also cardiac hemorrhages and a frank picture of hemorrhagic cystitis. In this context of visceral suffering, the slightly softer consistency of the main viscera is completely explainable despite the good state of preservation of the body. Moreover, being the subject young, in excellent physical health, and not following any pharmacological therapy, all the pathological alterations documented at histological examination are due to the toxic effects of PQ. Clinical, autopsy, histological, and toxicological data were fully consistent with the circumstantial information provided by the health workers involved in the rescue. Moreover, they all converge in demonstrating the very serious toxicity of PQ.

In this regard, it is fundamental to underline the importance of circumstantial data to guide toxicological investigations, as many substances, including PQ, are not covered by routine toxicological panels in both clinical and forensic settings. In general, when dealing with forensic cases in which the involvement of a toxic substance in the death event is present or suspected, it is customary to think directly of the forensic toxicological examination. This is correct from a procedural point of view, but it should never be forgotten that toxic substances are such as they are able to exert their damaging action directly or indirectly on biological tissues, leaving signs that only microscopic examination can bring out and correctly characterize in the pathophysiological reconstruction of the death event. From this derives the need to always associate histopathological with toxicological analysis, because, in addition to providing direct confirmation of the harmful effects produced by the substance, it can also bring to light specific peculiarities not yet known or rarely reported in literature, such as cardiac hemorrhage associated with the ingestion of caustic substances.^39,40^ This becomes even more crucial in cases of more or less prolonged survival periods after exposure to substances of toxicological interest, which, in the forensic field, are still poorly described, because, most of the times, the lethal event is immediate. In this context, PQ deaths are no exception, and the histological findings that emerge may be of clinical relevance in optimizing the treatment and management of these patients. Indeed, in this case, the finding of initial alveolar fibrosis was extremely relevant despite the patient's short survival. This demonstrates how the particularly damaging effect of PQ on the lungs can trigger the occurrence of early alveolar fibrosis. This finding should be considered a new element in the context of lung injury caused by PQ, as it has not been previously documented in literature. It only emerged as a result of a histological investigation in an extremely unusual case of short survival following massive PQ ingestion, contributing to the scientific enrichment of knowledge about this lethal poisoning.

To date, after more than 44 years from the first reports of human PQ poisoning, much remains to be clarified about its biological toxicity because of the few reported cases and the lack of complete knowledge of the lethal effects developed. Any scientific enrichment through different contributions can, therefore, be useful to improve knowledge about this lethal poisoning.
